# Asymptomatic trocar site hernias: An underestimated complication of laparoscopy

**DOI:** 10.4274/tjod.galenos.2020.70952

**Published:** 2020-10-02

**Authors:** Emin Üstünyurt, Fatma Nurgül Taşgöz, Sefa Tiğrak

**Affiliations:** 1 University of Health Sciences Turkey, Bursa Yüksek İhtisas Training and Research Hospital, Clinic of Gynecology, Bursa, Turkey; 2 University of Health Sciences Turkey, Bursa Yüksek İhtisas Training and Research Hospital, Clinic of Radiology, Bursa, Turkey

**Keywords:** Complications, laparoscopy, port site hernia, trocar, trocar site hernia

## Abstract

**Objective:**

To estimate the exact incidence of trocar site hernia (TSH) through sonographic examination and to evaluate the predisposing risk factors of TSH.

**Materials and Methods:**

Three hundred patients who underwent laparoscopic surgery for benign gynecologic indications were included in this study and called back for a follow-up visit. All patients underwent an ultrasound evaluation for the detection of TSH. Risk factors for TSH formation were investigated.

**Results:**

Twenty-five (8.3%) TSHs were diagnosed among 300 postoperative laparoscopies. The highest rate of TSH development among the surgeries was found in tubal ligation cases with 19%. Parity ≥3 [odds ratio (OR), 3.13; 95% confidence interval (CI): 1.21-8.09; p=0.018], and not closing fascia (OR: 6.74; 95% CI: 2.72-16.70; p<0.001) were statistically significant risk factors for the development of TSH in multivariate analysis.

**Conclusion:**

The prevalence of TSH is higher than previously reported, and ultrasonographic examination is adequate for detecting subclinical types of this complication.

**PRECIS:** When ultrasonography was used for diagnosis, it was observed that the frequency of trocar site hernia development was higher than previously reported.

## Introduction

Trocar site hernia (TSH) is defined as an incisional hernia (IH) occurring after laparoscopic procedures at the trocar incision site^(1)^. TSH is a rare surgical complication with an estimated incidence ranging between 0.6% and 5.2%^(2,3,4,5)^. However, because available data are based only on symptomatic patients and clinically diagnosed cases, the actual incidence is probably underestimated. The overall incidence might be higher if asymptomatic patients are routinely screened using a more effective diagnostic tool such as ultrasonography (USG).

Most TSHs are asymptomatic, but they can occasionally lead to severe morbidity and mortality, such as bowel strangulation and necrosis^(5)^. Thus, early detection of subclinical TSH before the occurrence of severe complications is essential. Many factors have been implicated as predisposing to TSH formation. Advanced age, obesity, diabetes mellitus and wound infection were identified as patient-related risk factors^(1,5,6,7)^. Moreover, trocar size, design, and insertion technique; port location; duration of surgery; fascial incision enlargement; and fascial closure were suggested as technical factors for the occurrence of TSH^(1,8,9,10)^.

When the literature on this subject is examined, it is seen that information about prevalence and risk factors of TSH, especially in the female population, is still limited and contradictory.

The primary objective of this study was to estimate the exact incidence of TSH by sonographic examination and to evaluate the predisposing risk factors of TSH.

## Materials and Methods

This study was conducted in University of Health Sciences Turkey, Bursa Yüksek İhtisas Training and Research Hospital, Clinic of Obstetrics and Gynecology. After receiving approval of the ethics committee, medical records of 491 patients who underwent laparoscopic surgery between May 2016 and July 2018 in our clinic were retrospectively scanned.

All patients undergoing a laparoscopic surgical procedure for benign gynecologic indications during the study period with a minimum follow-up of 12 months were included in the study. The exclusion criteria of the study were determined as follows: age <18 years old, previous history of midline laparotomy, pregnancy after the procedure, a subsequent abdominal surgical procedure except for IH repairs, use of the open technique or palmar point for entering the abdomen, conversion to laparotomy, malignancy, and refusal to give informed consent. Also, we excluded any patients who underwent laparoscopic surgery before or after the index procedure.

**Figure 1:**
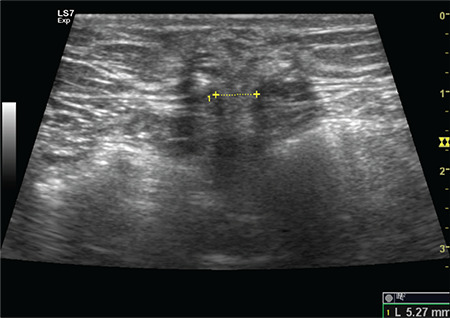
Ultrasound image showing herniation of omentum through the defect at the 10 mm trocar sit

Finally, 300 patients were included in the study and called back for a follow-up visit. The average time between the surgery and the patient’s sonographic evaluation with USG was 16 (range, 12-29) months. Written informed consent was obtained from all participants. All patients underwent a clinical examination. Furthermore, all participants underwent an ultrasonographic evaluation for the detection of TSH. The ultrasonographic diagnosis of TSH was defined as any discontinuation of the fascial layer (Figure 1). Clinical and sonographic examinations were performed by a single physician and a single radiologist. A 9L-D linear broad-spectrum transducer with the GE Health Care Logic S7 expert model USG was used.

Laparoscopic surgeries were performed by different surgeons working in our institution. Three or four-port sites were generally used for the procedures, one of which was 10/12 mm cutting trocar for the camera, and two or three were the lateral 5-mm cutting trocars. Initially, a 10 or 12 mm trocar was inserted with the closed technique to the umbilical location. Lateral trocars were placed approximately 6-8 cm from the midline and 4-5 cm above the symphysis. At our institution, the decisions for the type of abdominal entry (direct trocar or Veress needle), closure of umbilical port site (only skin or fascia and skin), and the placement of drain are based on the clinical judgment of the operative surgeon. If a drain was inserted, it is aimed to be removed within 1-2 days in cases without problems.

All patients received prophylactic antibiotherapy (1 to 2 g cefazolin was administered intravenously 15 to 60 minutes prior to skin incision). Skin sutures were removed at day 7-10.

For each patient enrolled in the study, the hospital and follow-up records were reviewed for the following data: pre-operative age in years, weight in kilograms and height in meters, body mass index (BMI) (calculated as weight in kilograms divided by height in meters squared), gravida, parity, type II diabetes [defined as blood glycemia >126 mg/dL and/or glycated hemoglobin (HbA1c) >7% and/or use of oral hypoglycemic agents and/or insulin], presence of chronic constipation, smoking status (defined as positive when actively smoking), type of abdominal access, fascia enlargement to remove material, closure of umbilical 10/12 mm trocar fascia, surgical duration, drain placement, and the presence of wound infection (defined as a positive culture and/or presence of infection according to the physician’s opinion).

### Statistical Analysis

Data were analyzed using the IBM SPSS V23 (SPSS, Inc., Chicago, IL). The Shapiro-Wilk test was used to examine the compatibility of data to normal distribution. The independent samples t-test was used to compare the parameters according to the presence of TSH. The chi-square test was used to evaluate the correlation between categorical data and TSH. Independent risk factors for the development of TSH were analyzed using univariate and multivariate logistic regression analysis. A p-value of <0.05 was considered as statistically significant.

## Results

There were 25 (8.3%) TSHs among 300 patients who underwent laparoscopic surgery. Of the 25 patients with TSH, 23 had a herniation at the umbilicus, and two had herniation at the extra-umbilical sites. The TSHs were asymptomatic in 23 patients (92% of all TSHs). Five were detected by physical examination and confirmed by ultrasound, whereas 18 patients had normal abdominal examination findings and were diagnosed as having TSH with USG.

Two patients have already undergone hernia repair surgery for symptomatic TSH in the 8^th^ and 10^th^ postoperative months, respectively. The first of these patients was a 31-year-old who underwent bilateral tubal ligation and developed wound infection in the postoperative period. She was admitted to the hospital eight months after surgery due to TSH containing omentum tissue in the umbilical region. The second case was a 38-year-old patient who underwent cystectomy. In this case, the cyst material was removed from the left lower quadrant, but fascia was not enlarged, and the drain was inserted from the same site. Ten months after the surgery, she was admitted to hospital with gastrointestinal symptoms and was immediately taken to the operating room due to the detection of intestinal herniation from the trocar site in the left lower quadrant.

The most frequent surgeries performed during the study period were cystectomy (33%), hysterectomy (25.3%), and tubal ligation (19.3%), respectively. The highest rate of TSH development among the procedures was found in tubal ligation cases with 19%. The rate of patients with a parity number of three or more was highest in the group undergoing tubal ligation (75.9%). Patient data and surgery types are presented in Table 1.

The gravida and parity of patients who developed hernia (3.4±1.58 vs 2.53±1.64; p=0.011 and 3±1.38 vs 2.15±1.53; p=0.008, respectively) were significantly higher. The fascia closure rate was significantly lower in patients with TSH compared with those without TSH (40% vs 82.2%; p<0.001, respectively). Characteristics of patients with and without TSH are presented in Table 2.

There were no statistically significant differences between patients with and without TSH regarding age (42.12±10.15 vs 40.64±11.11; p=0.520), BMI (28.44±4.26 vs 27.05±4.94; p=0.175), presence of comorbidities (p=0.410 for diabetes mellitus and p=0.665 for chronic constipation), preoperative hemoglobin values ​​(12.13±5.88 vs 11.89±1.44; p=0.838), and surgical duration (58.6±36.98 vs 65.05±33.41; p=0.360). Likewise, in terms of hernia development, no statistically significant difference was found between the following parameters: presence of previous surgery, smoking status, trocar entry technique, and fascial incision enlargement (p>0.05).

Parity ≥3 and the absence of fascia closure were statistically significant risk factors for the development of TSH in both univariate and multivariate analyses (Table 3). Wound infection was found to have a significant effect on TSH formation in multivariate analysis. There was no significant association with not placing a drain and TSH formation in the multivariate analysis.

## Discussion

Data on the frequency of TSHs show a wide distribution in the relevant literature. The lack of consensus regarding the definition of TSH among studies might influence the reported prevalence. TSH is a type of IH that occurs after laparoscopic surgery at the trocar incision site. IH was defined as any abdominal wall gap or defect in the proximity of the postoperative scar by most of the studies^(11,12,13)^. However, some of these studies included a protrusion of abdominal contents in the definition^(14)^. In this study, we defined hernia as any defect in the fascia layers in the trocar entry site because we aimed to detect all asymptomatic cases. Tonouchi et al.^(1)^ first classified three types of TSHs according to the cause and the onset time. The early-onset type of hernia occurs by the dehiscence of anterior and posterior fascial plane and peritoneum in the early postoperative period. The late-onset type of hernia occurs by the dehiscence of anterior and posterior fascial plane, with peritoneum providing the hernia sac; they appear several months after surgery. Small intestinal obstruction is not seen, and it manifests as an asymptomatic swelling. This special type of hernia is due to the dehiscence of the whole abdominal wall immediately after surgery, with intestine and/or omentum protruding without a sac^(1)^. All our cases except two symptomatic were thought to represent the late-onset type.

In many studies, the frequency of TSH is reported as between 0.6% and 5.2%^(2,3,4,5)^. According to these publications, the frequency of TSH of 8.3% in our study is significantly high. However, most of these studies focused on hernia cases that required symptomatic or surgical repair. Also, in studies involving asymptomatic cases, hernia diagnosis was made primarily with physical examination findings. In studies where additional imaging modalities were used for diagnosis other than physical examination, higher hernia identification rates were reported^(10,15,16)^. In the study of Christie et al.^(10)^ evaluating TSH development after robot-assisted urologic surgery, all patients underwent radiologic imaging (computed tomography), and the incidence of TSH was reported as 7.7%. In the literature, there are three studies that evaluated patients who underwent bariatric surgery with an imaging modality, and the incidence of TSH was approximately 24.5%^(17,18,19)^. In our study, the patients were invited to our clinic for re-evaluation after a minimum of 12 months and a maximum of 29 months after surgery. The incidence of TSH detection is significantly higher when the follow-up period is longer than 12 months, and when an imaging modality is used^(7)^.

Generally, there is worldwide variability in the abdominal entry techniques as well as port site closures, and our institution is no different. Some surgeons prefer the Veress needle, whereas others perform the direct trocar technique. Likewise, there is no consensus among surgeons regarding the closure of trocar entry points. In many studies, it is emphasized that leaving the fascia open is the most important factor in the development of TSH, and closure of the fascia is recommended in trocar site incisions of 10 mm and over^(1,5,20,21)^. In the first systemic review of TSH, Tonouchi et al.^(1)^ indicated that surgical technique-related factors rather than patient-related factors were of primary importance in the formation of TSH, and reported that large trocar diameter, open facial defects, and stretching of port sites were strictly related to TSH formation. They suggested the closure of the facial defects of umbilical or extra umbilical areas where trocar diameters of 10 mm and over were used as well as the closure of the fascial defect in the case of active manipulation from the 5-mm port during lengthy procedures. In a systemic review compiled by Helgstrand et al.^(6)^ 96% of TSHs were reported to occur in trocar locations with 10 mm and larger diameter trocars, and 82% were in the umbilicus; it was suggested to close the facial defects of trocars with a diameter of 10 mm and over. Also, in many studies, it has been reported that 12-mm cutting trocars caused higher rates of hernia formation compared with 10-mm bladeless trocars^(22,23)^. In our study, 92% of the detected hernias occurred at the port entry points of 10-12 mm trocars. Moreover, in more than half (60%) of our patients who developed hernias, fascial closure was not performed. Our findings also support the literature suggesting the closure of the trocar sites over 10 mm. In the systemic review published by Karampinis et al.^(7)^ in 2019, contrary to expectations, a higher incidence of TSH was found in studies that routinely performed fascial closure in patients who underwent laparoscopic bariatric procedures. However, this result did not reach statistical significance. In our study, we also observed that fascial closure was performed in 40% of hernia cases. This result may be due to the fact that the surgeries were performed by different surgeons with different experiences and skills; therefore, fascia closure may not be effectively performed.

The role of sex in the development of TSH is conflicting in the literature^(5,6,8)^. It is evident that a condition that may lead to laxity and fascial defects, especially in the anterior abdominal wall, such as pregnancy and labor, may be an important risk factor for hernia development. The data on the effect of parity on hernia development in the literature are minimal. It is known that relaxation and damage occur in the abdominal wall and fascial structures due to childbirth^(8)^. This damage in the umbilical region may cause fascial defects in the following years. In a study involving 2.100 cases, 18% of patients had a fascial defect in the anterior abdominal wall during laparoscopy^(24)^. Considering factors such as age, BMI, and surgical time, tubal ligation cases were in a low-risk group for hernia development. However, TSH development rate per surgery was found to be significantly higher in these patients in our study. This finding cannot be explained only by the low fascia closure rate. We believe that the high parity number in tubal ligation cases indicates that parity is an important risk factor for TSH development.

To the best of our knowledge, there are no studies examining the relationship between drain insertion and TSH development in the literature. In this study, we aimed to evaluate whether drain insertion decreased the development of TSH by reducing intraabdominal pressure. Although drain insertion resulted in fewer cases of TSH in our study, this finding did not reach statistical significance. On the other hand, drain use may contribute to the formation of hernia by causing infection. Evidence regarding the relationship between closed suction drain (CSD) and surgical site infection (SSI) in the obstetrics and gynecology literature is conflicting^(25)^. A few studies suggested an increased risk of SSI associated with drain placement but usually associated with open drainage and not the use of CSD^(25)^. We also believe that short-term closed drainage does not play an important role in the development of infection. The data of both this study and current studies are insufficient to determine the effect of drain insertion on TSH development. We believe that prospective randomized studies on this subject are necessary to clarify this issue.

Although many studies have reported that age and BMI might be risk factors for the development of TSH, such a relationship has not been reported in other publications^(5,6,7,8,26)^. Especially in studies evaluating patients who underwent bariatric surgery, obesity has been reported to be an important risk factor for TSH due to the high intraabdominal pressure and the full thickness of the preperitoneal area, which causes a challenge for closure and increase in wound infection^(17,18)^. Likewise, being aged over 60 or 70 years has been reported to be associated with increased TSH^(8,15,27)^. Our study population was relatively younger and had a lower weight average than the published literature reporting increased risk, and no relationship was found between age or BMI and TSH development. It could be explained by the fact that our study group did not have the extreme values ​​stated in the literature regarding the mentioned factors.

Contrary to previous studies reporting the association between surgical duration and TSH, surgical duration did not differ between the patients who had TSH^(8,27)^. Moreover, the highest TSH rate was observed in tubal ligation cases with the shortest surgical duration. The fact that tubal ligation cases had higher values ​​in terms of parity, which was determined as an important risk factor for TSH also in this study, may have masked the effect of surgical time.

In a recently published study, excessive manipulation of the trocar site to remove specimens during surgery was reported to be an important risk factor for TSH formation^(28)^. The authors also suggested avoiding conditions that increased abdominal pressure such as coughing within 2 weeks after surgery. In our study, the port site for specimen removal was not mentioned in the surgical notes. Likewise, we did not have any data on the exposure of patients to conditions that might increase intra-abdominal pressure in the early postoperative period.

### Study Limitations

Our study has limitations. The missing data in the medical records is the structural limit of our study; we were unable to control or evaluate many factors that could affect hernia development, such as the use of different brands and sizes (10 or 12 mm) of trocars. The performed surgeries have technical variations because different surgeons performed the procedures. Differences in entering the abdomen (e.g. trocar insertion angle, excessive manipulations) and closure techniques may have influenced the outcomes. Also, the structure and the size of our sample may not be sufficient to determine the effect of individual factors, such as age, BMI, wound infection, and comorbidities.

The strength of our study is the diagnostic method we use in diagnosing TSH. Regardless of the physical examination findings, the evaluation of all patients using USG enabled us to diagnose all asymptomatic or subclinical cases. Thus, we believe that the frequency of TSH detected in our study reflects the true prevalence of this complication. Also, contrary to the relevant literature, which mostly includes general surgery and urology patients, the data of our study, which consists of only female cases, can provide predictions about the risk of TSH in common basic gynecologic procedures. Another important finding of our study is the high rate of TSH in young women with high parity. We believe that the high parity, which has not been sufficiently evaluated in studies published to date, should be considered as an important risk factor for hernia development regardless of the type of surgery.

## Conclusion

Our findings suggest that the prevalence of TSH is higher than previously reported, and an ultrasonographic examination is sufficient for identifying subclinical types of this complication.

## Figures and Tables

**Table 1:**
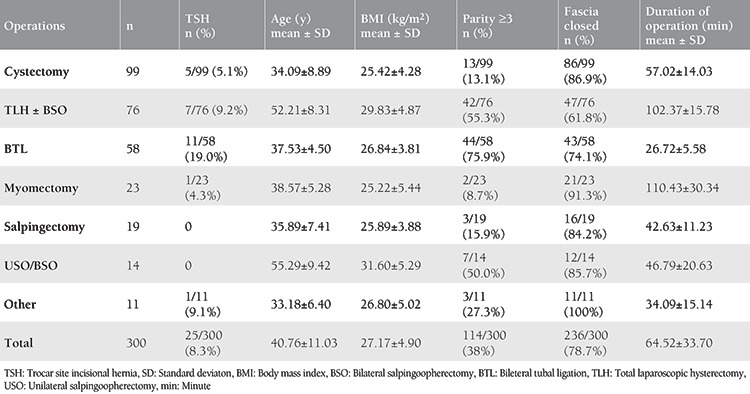
Patient data and types of surgery

**Table 2:**
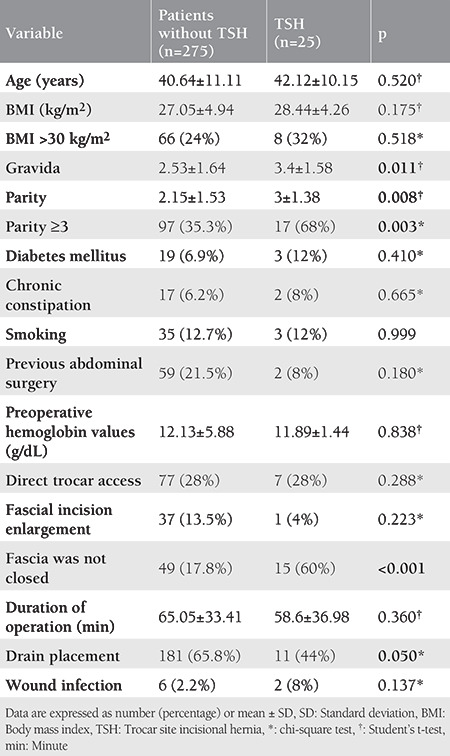
Characteristics of patients with and without trocar site incisional hernia

**Table 3:**
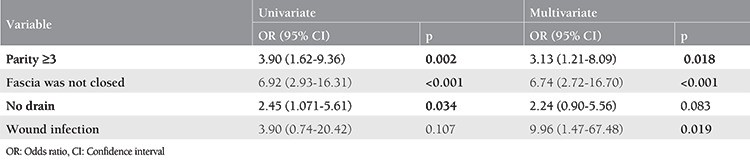
Univariate and multivariate logistic regression analysis for development of trocar site incisional hernia
